# The Antibacterial Action of Safranin and Gentian Violet

**DOI:** 10.5041/RMMJ.10475

**Published:** 2022-07-31

**Authors:** Falah AL-Khikani, Aalae Ayit

**Affiliations:** 1 Medical Technique Department, College of Medical Technique, The Islamic University, Babylon, Iraq; 2 Department of Microbiology, Al-Shomali General Hospital, Babylon, Iraq

**Keywords:** Antimicrobial agents, dyes, gentian violet, *Pseudomonas aeruginosa*, safranin, *Staphylococcus aureus*

## Abstract

**Background:**

The increasing resistance of many bacterial pathogens against antibiotic measures urgently requires new or repurposing therapeutic strategies. Gentian violet is a triarylmethane dye used as a histological stain and for Gram’s method of classifying bacteria. It also exerts an antimicrobial effect against certain pathogens, especially dermatological infections. Safranin is the most popular counterstain used in medical laboratories due to its low cost and safe laboratory usage. However, few studies have been conducted on the antimicrobial activity of safranin.

**Objective:**

With the growing prevalence of multidrug-resistant bacteria, this study aimed to evaluate the antibacterial efficacy of gentian violet and safranin against multidrug-resistant *Staphylococcus aureus* (*S. aureus*) and *Pseudomonas aeruginosa* (*P. aeruginosa*).

**Methods:**

All tested bacteria were multidrug-resistant (MDR) bacteria isolated from skin infections (abscesses and wounds). Using gentian violet and safranin, antibacterial effects were studied using the well-diffusion method against 20 samples of clinically isolated bacteria, 10 diagnosed as *S. aureus*, and 10 as *P. aeruginosa*. Bacteria were diagnosed using the VITEK 2 automated system (bioMérieux, Marcy-l’Étoile, France). Iodine served as the control agent, since both Gram-positive and Gram-negative bacteria are sensitive to it.

**Results:**

Gentian violet dye has been shown to be 100% sensitive to both Gram-positive and Gram-negative bacterial isolates. Although safranin also had high sensitivity (100%) to *S. aureus* isolates, its sensitivity to *P. aeruginosa* was only 20%. *Staphylococcus aureus* was more resistant to iodine (40% sensitivity) compared to *P. aeruginosa*, which was 100% sensitive to iodine.

**Conclusions:**

Gentian violet and safranin are low-cost and better tolerated topical agents that have potential for use in dermatological applications. Gentian violet had good antibacterial activity against both Gram-positive and Gram-negative bacteria, making it useful for treating bacterial skin pathogens such as *S. aureus* and *P. aeruginosa* especially for MDR bacteria. While safranin has good efficacy against Gram-positive bacteria (*S. aureus*), its effect against Gram-negative bacteria (e.g. *P. aeruginosa*) is poor.

## INTRODUCTION

The Gram stain, a procedure invented by Hans Christian Gram in 1884, uses differential staining with a gentian violet–iodine complex and a safranin counterstain to discriminate between Gram-positive and Gram-negative bacteria. After being exposed to alcohol, the cell walls of Gram-positive organisms maintain this complex and look purple, whereas the cell walls of Gram-negative organisms decolorize and become pink.^1^

Throughout the first half of the twentieth century, gentian violet was widely adopted for use in a variety of diseases including trench mouth, thrush, impetigo, burns, pinworm, and cutaneous and systemic fungal infections.^2^–^3^ Claims of gentian violet efficacy during this time period are difficult to ascertain, given that the composition of gentian violet dyes varied and the authors did not always describe the precise solutions used in their publications. Its use lost popularity with physicians with the discovery and mass manufacture of sulfa medicines and penicillin in the 1940s, and scientific research shifted to the development of novel antibiotic families. However, with the rise of antibiotic resistance there has been a recent resurgence of interest in gentian violet treatment for anti-sepsis and other purposes. Recent research on its mechanisms of action has broadened its potential use in dermatology. Gentian violet is widely accessible, affordable, and simple to use, with few negative effects.

Gentian violet seems to have two unique modes of action that contribute to its therapeutic use. It inhibits the NADPH oxidase complex, which includes Nox1,2, and 4, in mammalian cells, resulting in decreased superoxide generation. Gentian violet can form a covalent adduct with thioredoxin-2 (TRX-2) in bacteria, fungi, and parasites. The discovery that gentian violet inhibits NADPH oxidase revealed the effect of gentian violet on both host and pathogenic organisms, which could be leveraged to improve anti-angiogenesis and tumor immunity in the twenty-first century.^4^ Antibacterial, antifungal, antihelminthic, antitrypanosomal, antiangiogenic, and anticancer activities are all found in gentian violet.^5^,^6^ Burn wounds are treated with gentian violet in resource-constrained circumstances.^7^

Gentian violet’s long history of topical and systemic usage, especially in the prevention of illness, as well as its stability—particularly at room temperature for long periods of time—has made it a cornerstone of dermatologic therapy in developing nations. Several considerations, including the rise of antibiotic resistance and the widespread use of catheters and indwelling devices, imply that gentian violet should be used more widely in industrialized countries as well.

Initial studies performed with mice showed an increased rate of hepatocellular carcinoma in mice fed large doses of 300 to 600 parts per million gentian violet.^8^ Despite nearly a century of use, not a single case of cancer has been definitively associated with gentian violet use.^9^

Safranin (also referred to as safranin O or basic red 2), a less expensive and safer plant histology stain, could potentially be utilized for human tissues since it provides equivalent or better accuracy in the diagnosis of frozen sections of basal and squamous cell carcinomas.^10^

The biological stain safranin is used in histology and cytology. Certain staining techniques utilize it as a counterstain, turning cell nuclei red. In both Gram and endospore staining, this is the standard counterstain. It may be used to identify cartilage, mucin, and mast cell granules, among other things.^11^

In light of the recent emergence of a significant incidence of bacterial resistance, repurposing of these antimicrobial medicines merits serious consideration.^12^–^14^ To that end this study tested the antibacterial efficacy of gentian violet and safranin against the multidrug-resistant organisms *Staphylococcus aureus* (*S. aureus*) and *Pseudomonas aeruginosa* (*P. aeruginosa*).

## MATERIALS AND METHODS

Both *S. aureus* and *P. aeruginosa* are commonly encountered pathogens in wounds. Hence, clinical isolates from patient wounds or abscesses were obtained following receipt of verbal informed consent. For the purposes of this study, we selected 20 isolates: 10 Gram-positive *S. aureus* isolates from abscesses, and 10 Gram-negative *P. aeruginosa* isolates from abscesses and wound swabs. The VITEK 2 automated system (bioMérieux, Marcy-l’Étoile, France) was used to identify bacterial pathogens after growth on artificial media.

### Dye Preparation

Gentian violet stock solution was prepared by dissolving 20 g of crystal violet in 100 mL ethanol. To create an oxalate stock solution, 1 g of ammonium oxalate was dissolved in 100 mL water. The working solution was made up of 1 mL crystal violet stock solution, 10 mL water, and 40 mL of oxalate stock solution, which was stored in a drop vial. The final concentration was gentian violet 0.2%.

The stock solution of safranin was created by dissolving 2.5 g of safranin in 100 mL of 95% ethanol. The working solution was one part stock solution in five parts water.^15^ The final concentration was safranin 0.025%.

### Antibacterial Activity Screening

Standard agar well diffusion was used to determine the antibacterial activity *in vitro*. Petri-dishes were prepared with ~25 mL of autoclaved nutritional agar poured onto sterile plates and allowed to set. The surface of each plate was then drilled with a sterile cork borer (6 mm) and three wells punched out. A total of 50 μL of standardized test organism culture (adjusted to 0.5 McFarland 107 CFU/mL) was placed on the agar plate. The wells were then filled with 50 μL of dye; 50 μL of iodine was used as a positive control. The seeded Petri-dishes were incubated at 37°C for 24 hours before measuring the inhibition zone. Calculations of zone inhibition were made were according to the Clinical and Laboratory Standards Institute: weak antibacterial activity, zone <10 mm; moderate antibacterial activity, zone 10–13 mm; strong antibacterial activity, zone >13 mm.^16^,^17^

Ceftriaxone (30 mg), ceftazidime (30 mg), cefotaxime (30 mg), gentamicin (10 mg), amikacin (30 mg), ciprofloxacin (5 mg), imipenem (10 mg), penicillin (10 mg), erythromycin (15 mg), trimethoprim/ sulfamethoxazole (25 mg), vancomycin (30 mg), and meropenem (10 mg) were tested for antimicrobial susceptibility using Kirby–disc-diffusion Bauer’s technique (10 mg). Bacteria were classified as multidrug-resistant (MDR) if they were resistant to more than two classes of antibiotics.

### Data Analysis

For both *S. aureus* and *P. aeruginosa*, the numbers of clinical isolates showing weak, moderate, and strong activity were noted. For statistical analysis, SPSS software 14.0 (SPSS Inc., Chicago, IL, USA) was used. Differences in the rate of strong activity between iodine, gentian violet, and safranin were evaluated using chi-square test for independence. Differences between the radii of the zones of inhibition were analyzed with the Mann–Whitney test.

### Ethics

This study was approved by the Ethical Committee of the Babylon Health Directorate (June 3, 2021; #37815). Before collecting samples, patients or their parents (if the patient was a minor) provided verbal consent. Standard health and safety precautions were followed when acquiring the samples.

## RESULTS

Ten Gram-positive *S. aureus* isolates from abscesses and 10 Gram-negative *P. aeruginosa* isolates collected from pus and wound specimens were analyzed. All of the isolates were MDR (Table 1).

**Table 1:** Antibiotic Resistance of Bacterial Isolates in This Study.

Antibiotics	*Pseudomonas aeruginosa* (Resistance %)	*Staphylococcus aureus* (Resistance %)
Amikacin	50%	60%
Amoxicillin	100%	100%
Cefotaxime	80%	100%
Ceftazidime	90%	90%
Ceftriaxone	80%	90%
Ciprofloxacin	60%	70%
Erythromycin	100%	80%
Gentamicin	90%	50%
Imipenem	50%	30%
Meropenem	50%	30%
Penicillin	100%	100%
Trimethoprim/Sulfamethoxazole	100%	80%
Vancomycin	100%	50%

Both gentian violet and safranin showed strong antibacterial activity against all of the tested *S. aureus* isolates (Table 2). Both gentian violet and safranin fared better compared to iodine, which showed strong antibacterial activity in only five of the 10 *S. aureus* isolates (*P*=0.003). The median (IQR) radius of the zone of inhibition was 24 mm (21.75–25.75 mm) for gentian violet and 18 mm (17–20.25 mm) for safranin. There was a significant difference between the radii of the zones of inhibition created by the two stains (*P*=0.001).

**Table 2:** The Inhibition Zones (in mm) for Gentian Violet, Safranin, and Control (Iodine) Dyes in All 20 Samples.

Bacteria	Gentian Violet	Safranin	Iodine
*Staphylococcus aureus*	25	20	17
*Staphylococcus aureus*	23	17	12
*Staphylococcus aureus*	21	20	13
*Staphylococcus aureus*	22	18	12
*Staphylococcus aureus*	23	17	15
*Staphylococcus aureus*	25	18	17
*Staphylococcus aureus*	21	18	11
*Staphylococcus aureus*	25	17	17
*Staphylococcus aureus*	29	21	15
*Staphylococcus aureus*	28	22	11
*Pseudomonas aeruginosa*	26	0	26
*Pseudomonas aeruginosa*	16	0	22
*Pseudomonas aeruginosa*	16	0	26
*Pseudomonas aeruginosa*	19	0	20
*Pseudomonas aeruginosa*	27	18	18
*Pseudomonas aeruginosa*	28	0	24
*Pseudomonas aeruginosa*	21	18	19
*Pseudomonas aeruginosa*	22	0	20
*Pseudomonas aeruginosa*	15	0	17
*Pseudomonas aeruginosa*	23	0	22

Inhibition zones: Weak <10 mm; moderate 10–13 mm; strong >13 mm.

Unlike the case of *S. aureus*, only gentian violet and iodine showed strong antibacterial activity against all 10 *P. aeruginosa* isolates tested (Table 2). Compared to gentian violet and iodine, only two of the 10 *P. aeruginosa* isolates proved to be sensitive to safranin (*P*<0.001). The median (IQR) radius of the zone of inhibition was 21.5 mm (16–26.25 mm) for gentian violet and 21 mm (18.75–24.5 mm) for iodine. The difference between the radii of the zones of inhibition created by the two stains was not significant (*P*=0.98).

## DISCUSSION

This study found that gentian violet has a high rate of effectiveness against both Gram-positive and Gram-negative bacteria. Bakker et al. had already explored gentian violet as an antimicrobial for dermatological illness in 1992.^18^ After using gentian violet and a similar triphenylmethane dye to stain five bacterial species (*Streptococcus* A and B, *Proteus*, *P. aeruginosa*, and *S. aureus*) and *Candida albicans*, a low critical concentration of gentian violet was shown to be very efficient against *Candida*, *Streptococcus*, and *Staphylococcus* species; it was also moderately effective against Gram-negative bacteria.

Because of its ability to penetrate the bacterial cell wall and covalently link to proteins, gentian violet is very efficient against Gram-positive bacteria. Due to its inability to permeate the lipids surrounding the cell membrane, gentian violet is significantly less efficient against Gram-negative bacteria and *Mycobacterium*. The Gram stain, which has been in clinical use for over a century, is based on this principle.^4^

In one study, researchers separated 38 consecutive individuals with acute eczema colonized with *S. aureus* into three treatment groups; only gentian violet 0.3% had any anti-*Staphylococcus* efficacy *in vitro*. Gentian violet was also observed to dramatically reduce *S. aureus* density in both afflicted and unaffected skin after 4 days, as well as the clinical severity of eczema.^19^ Gentian violet has also been shown to be less efficient against Gram-negative bacteria in early trials.^20^ It destroys *Pseudomonas* biofilms *in vitro*, according to a recent study.^21^

In the largest study of its kind, conducted by the United States Food and Drug Administration (FDA), large doses of gentian violet were fed to rats over their lifetime. After 2 years, an increase in thyroid cancer was seen.^22^ Given that gentian violet is a nicotinamide adenine dinucleotide phosphate oxidase inhibitor, it most likely also inhibits the structurally similar thyroid peroxidase, causing hypothyroidism and feedback stimulation of thyroid-stimulating hormone from the pituitary gland, causing the replication of thyroid cells.^23^ In early attempts to treat bacterial sepsis, and as an antiprotozoal for strongyloidiasis and Chagas disease, humans were exposed to systemic doses of gentian violet, which was effective in treating these diseases.^4^ Therefore, while gentian violet is not free of side effects, researchers believe that topical gentian violet is safe for use in humans.^9^

In addition to its excellent recycling abilities, the pseudo-first order and intraparticle diffusion models play a role in the process of gentian violet adsorption resulting in a strong inhibitory effect on *Escherichia coli* growth.^24^ Gentian violet has been used to successfully treat MRSA in otitis media^25^ as well as nasal carriage of MRSA outside of dermatology.^26^ Hence, it is again emerging as a viable therapy option for MRSA infections in light of rising prevalence and evolving resistance to current medicines.

Our study found safranin to be 100% effective against Gram-positive bacteria (*S. aureus*), but it had only 20% efficacy against Gram-negative bacteria (*Pseudomonas* A). Only a few studies have looked at safranin’s antibacterial activity against microorganisms. Photodynamic therapy with safranin had a pronounced antibacterial effect on *Fusobacterium nucleatum* and *Porphyromonas gingivalis*, and *Streptococcus gordonii* was fully eradicated.^27^ Although gentian violet staining is routinely used to quantify biofilm growth, it has been linked to toxicity. Safranin, on the other hand, is a non-toxic stain that may be utilized in clinical settings to safely treat a variety of diseases.^28^

## CONCLUSIONS

The increasing prevalence of MDR bacteria is a major concern worldwide. This study highlights the significant antibacterial activity of some dyes, specifically gentian violet and safranin, against MDR pathogens. The results support the potential for using these dyes to manage patients in order to control the spread of MDR pathogens and the nosocomial infections in hospitals, particularly for abscess and wound infections, which can be treated topically, making them safer to use.

Gentian violet is potentially useful for treating bacterial abscess or wounds for both Gram-positive and Gram-negative bacteria, especially those with MDR isolates. Safranin would be useful for treating Gram-positive bacteria such as *S. aureus*, but is much less effective against Gram-negative bacteria. This study also found that iodine has greater efficacy against Gram-negative bacteria compared to Gram-positive bacteria, which may be highly iodine-resistant.

## Abbreviations

FDA: Food and Drug Administration
MDR: multidrug-resistant
MRSA: methicillin-resistant *Staphylococcus aureus*
*P. aeruginosa*: *Pseudomonas aeruginosa*
